# MM120 (lysergide D-tartrate) Receptor Binding and Evaluation in the Rat Following 13-weeks of Administration

**DOI:** 10.1192/j.eurpsy.2026.11606

**Published:** 2026-07-31

**Authors:** J. Tripp, G. Smagin

**Affiliations:** Non-Clinical, MindMed, New York, United States

## Abstract

**Introduction:**

MM120 (lysergide D-tartrate, LSD, CAS 32426-57-6) is under development as a potential treatment for generalized anxiety disorder and major depressive disorder. MM120 may elicit profound lasting psychological changes through interaction with brain 5-HT_1A_, _1B_, _2A_, _2C_, 6 and 7 receptors, among others, while the complete mechanism is not clear.

**Objectives:**

The study was performed to determine receptor occupancy and receptor density for 5-HT_2A_ and 5-HT_1A_ receptor in rats after chronic administration of MM120.

**Methods:**

Rats were administered MM120 (0, 0.5, 2.0, 6.0 mg/kg) daily or weekly (6.0 mg/kg) for 13-weeks and tested for toxicity, neurological safety (functional observational battery [FOB]) or physiological states. 5-HT_2A_ and 5-HT_1A_ ex vivo receptor occupancy (RO) was measured by incubation of brain sections with [^3^H]8-OH-DPAT or [^3^H]Ketanserin and quantification of binding. Receptor density was then measured in cortical and hippocampal washed membranes.

**Results:**

Chronic dosing showed no changes in body temperatures or FOB at week 13. Body weight changes were lower for 6 mg/kg daily group, but not weekly. Receptor density showed significantly lower binding of [^3^H]Ketanserin at 6 mg/kg daily (p < 0.05), compared to vehicle and 6 mg/kg weekly groups, indicating a lower 5-HT_2A_R density. 5-HT_1A_R density revealed with [^3^H]8-OH-DPAT binding was significantly lower in 6 mg/kg daily (p < 0.05) group, while other groups were unaffected. in vitro receptor binding and functional activity was evaluated and compared to other drugs of abuse. Chronic administration of MM120 at all doses significantly downregulated 5-HT_2A_Rs in cortex and hippocampus, while 5-HT_1A_Rs were affected only at the 6 mg/kg dose.

**Image 1:**
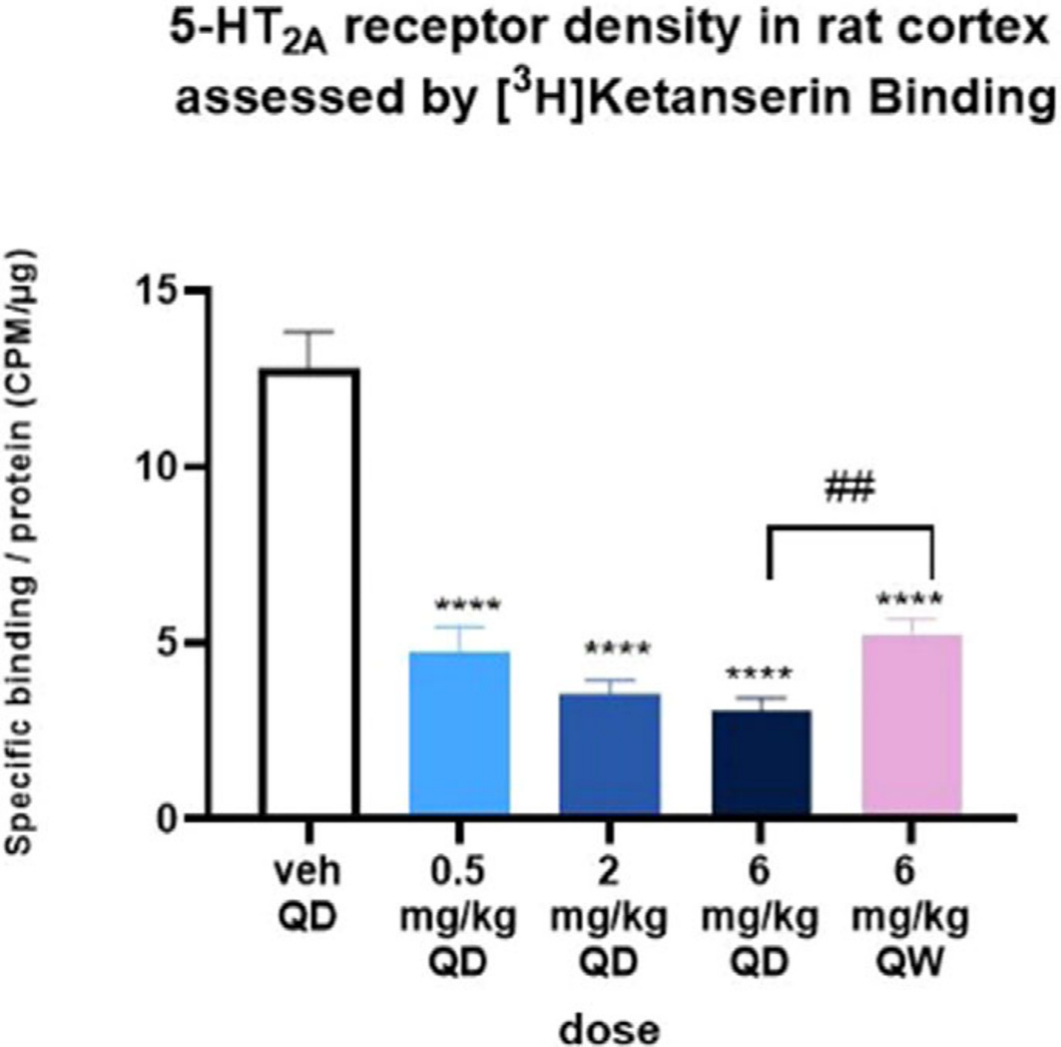
Image 1: Long description.

**Image 2:**
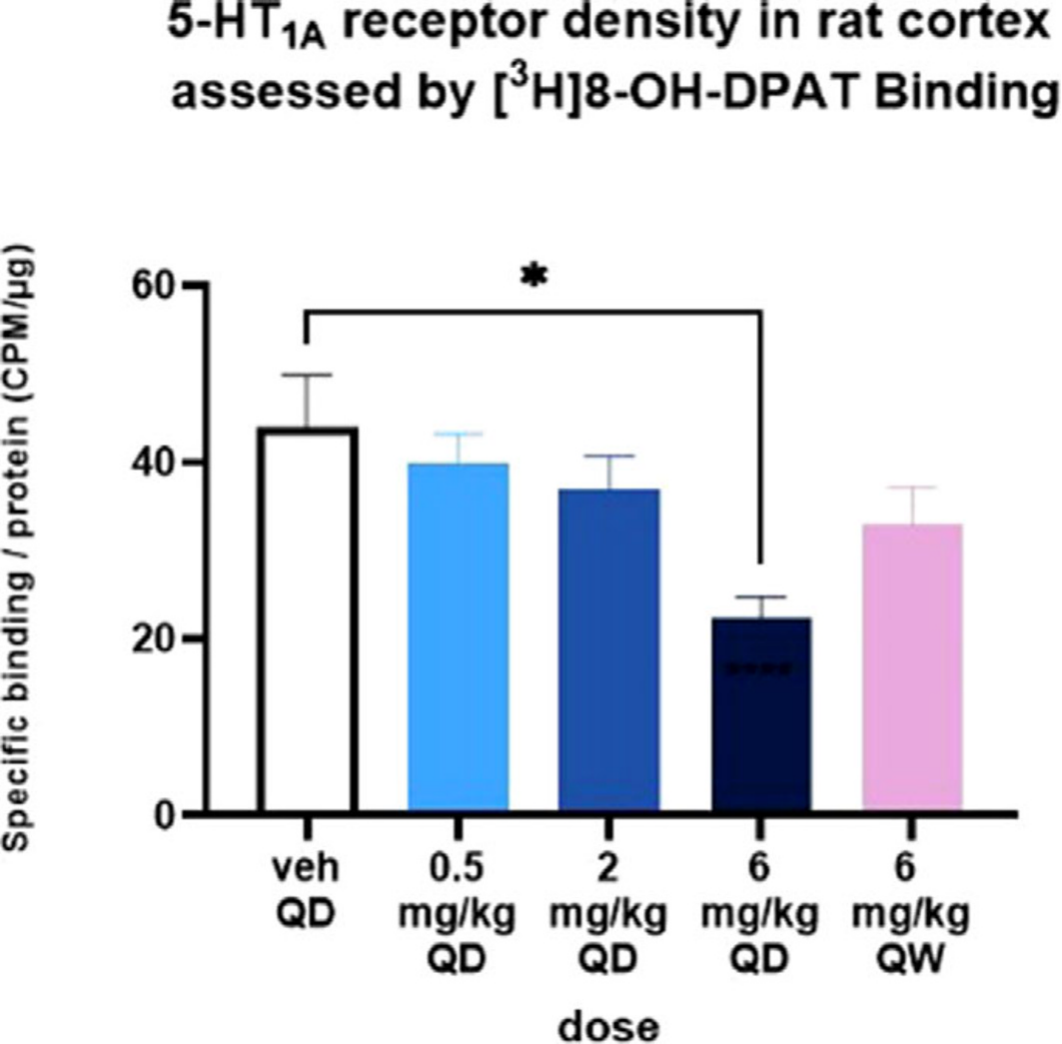
Image 2: Long description.

**Conclusions:**

The study demonstrates that 5-HT_2A_R downregulation may be responsible for the tolerance development after the repeated dosing and reduced abuse potential of MM120.

**Disclosure of Interest:**

J. Tripp Shareolder of: 100%, Employee of: 100%, G. Smagin Shareolder of: 100%, Employee of: 100%

## Long descriptions

### Image 1: Long description

The y-axis is labeled Specific binding / protein C P M / micro g with a scale from 0 to 15. The x-axis is labeled dose and contains five categorical bars.

* The first bar on the left is a white bar labeled veh Q D, showing a value of approximately 13.

* The second bar is light blue labeled 0.5 mg/kg Q D, showing a significant decrease to approximately 5, marked with four asterisks.

* The third bar is medium blue labeled 2 mg/kg Q D, showing a further decrease to approximately 4, marked with four asterisks.

* The fourth bar is dark blue labeled 6 mg/kg Q D, showing the lowest binding at approximately 3, marked with four asterisks.

* The fifth bar is pink labeled 6 mg/kg Q W, showing a value of approximately 5, marked with four asterisks.

A bracket with two hash symbols connects the 6 mg/kg Q D and 6 mg/kg Q W bars, indicating a statistical comparison between daily and weekly dosing at that concentration. All treatment bars show error bars extending above the main bar body.

Navigate back to Image 1:.

### Image 2: Long description

The bar graph is titled 5-H T sub 1 A receptor density in rat cortex assessed by [super 3 H] 8-O H-D P A T Binding.

* The y-axis is labeled Specific binding / protein (C P M / micro g) with a scale from 0 to 60 in increments of 20.

* The x-axis is labeled dose and contains five categories from left to right:

1. veh Q D: A white bar reaching approximately 44 units.

2. 0.5 mg/kg Q D: A light blue bar reaching approximately 40 units.

3. 2 mg/kg Q D: A medium blue bar reaching approximately 37 units.

4. 6 mg/kg Q D: A dark blue bar reaching approximately 22 units. This bar has four small asterisks inside it.

5. 6 mg/kg Q W: A pink bar reaching approximately 33 units.

All bars include vertical error bars. A horizontal bracket with a single asterisk above it connects the veh Q D bar and the 6 mg/kg Q D bar, indicating a statistically significant difference between these two groups.

Navigate back to Image 2:.

